# Warming and Nitrogen Addition Alter Photosynthetic Pigments, Sugars and Nutrients in a Temperate Meadow Ecosystem

**DOI:** 10.1371/journal.pone.0155375

**Published:** 2016-05-12

**Authors:** Tao Zhang, Shaobo Yang, Rui Guo, Jixun Guo

**Affiliations:** 1 Institute of Grassland Science, Northeast Normal University, Key Laboratory of Vegetation Ecology, Ministry of Education, Changchun, China; 2 Institute of Environment and Sustainable Development in Agriculture, Chinese Academy of Agricultural Sciences, Key Laboratory of Dryland Agriculture, Ministry of Agriculture, Beijing, China; University of California Davis, UNITED STATES

## Abstract

Global warming and nitrogen (N) deposition have an important influence on terrestrial ecosystems; however, the influence of warming and N deposition on plant photosynthetic products and nutrient cycling in plants is not well understood. We examined the effects of 3 years of warming and N addition on the plant photosynthetic products, foliar chemistry and stoichiometric ratios of two dominant species, i.e., *Leymus chinensis* and *Phragmites communis*, in a temperate meadow in northeastern China. Warming significantly increased the chlorophyll content and soluble sugars in *L*. *chinensis* but had no impact on the carotenoid and fructose contents. N addition caused a significant increase in the carotenoid and fructose contents. Warming and N addition had little impact on the photosynthetic products of *P*. *communis*. Warming caused significant decreases in the N and phosphorus (P) concentrations and significantly increased the carbon (C):P and N:P ratios of *L*. *chinensis*, but not the C concentration or the C:N ratio. N addition significantly increased the N concentration, C:P and N:P ratios, but significantly reduced the C:N ratio of *L*. *chinensis*. Warming significantly increased *P*. *communis* C and P concentrations, and the C:N and C:P ratios, whereas N addition increased the C, N and P concentrations but had no impact on the stoichiometric variables. This study suggests that both warming and N addition have direct impacts on plant photosynthates and elemental stoichiometry, which may play a vital role in plant-mediated biogeochemical cycling in temperate meadow ecosystems.

## Introduction

Global warming caused by the emission of greenhouse gases has increased significantly [1]; the global surface temperature has increased by 0.2°C per decade in the past 30 years [2], and it will continue to increase in the future [3]. Previous studies have shown that the elevated temperature has large impacts on carbon (C) and nutrient cycles in terrestrial ecosystems [4–5]. Elevated temperature also affects plant species establishment, community composition, and the productivity of terrestrial ecosystems [6–7]. Meanwhile, changes in plant community composition can alter ecosystem structure and function [8]. Therefore, understanding the response of plant growth and nutrient status to simulated climatic warming is important to predict terrestrial ecosystem community composition and nutrient cycling response to global warming in the future. Many studies have demonstrated that global warming directly influences plant physiological processes by affecting foliar photosynthetic performance [9–11] and stoichiometry [12–13], and it can also affect plant growth indirectly by influencing the utilization of water and nutrients [14]. However, the response of leaf photosynthetic products and C:N:P stoichiometry to global warming are far from clear, especially in natural ecosystems.

Anthropogenic nitrogen (N) deposition, which can affect carbon cycles, floristic diversity, is another important global threat to ecosystems [15–18]. China is one of the three largest N deposition regions [19]. Some areas in northern China received >2.0 g N m^-2^ yr^-1^ in the 2000s [20], and N deposition has a negative influence on agriculture and natural ecosystems [21]. An increasing number of studies have found that N deposition strongly reduces plant diversity, forb abundance and species richness [15,22–24]. In most N-limited ecosystems, increasing N availability leads to higher photosynthetic rates [11,25] and plant growth, increased N and P concentrations [26] and higher primary productivity [27]. However, the response of plant photosynthetic product accumulation and stoichiometry to N deposition is not well understood.

The increases in N deposition and global warming will continue simultaneously in the future [3]. Most previous studies have concentrated on the effects of N addition or warming alone on plant growth, community composition and productivity, etc. [15]. Although several studies examined the influence of warming and N addition on plant community net ecosystem CO_2_ exchange and photosynthetic responses [11], the interactive effects on plant leaf photosynthetic products and C:N:P stoichiometry remain unclear.

The Songnen grassland is a typical meadow steppe that is located along the eastern edge of the Eurasian continent. The temperature in this region will continue to increase by 2.8–7.5°C in the next 100 years [3]. Nitrogen deposition has significantly altered net ecosystem CO_2_ exchange [4], soil nutrients [5], plant community composition and productivity [28] in this meadow ecosystem. However, the effects of warming and N addition on plant leaf photosynthetic products and C:N:P stoichiometry are not clear. To determine the potential effects of climate warming, N deposition and their interaction on plant leaf photosynthetic products and C:N:P stoichiometry, we conducted a manipulative field experiment with increased temperature and N addition in the Songnen meadow steppe in northeastern China; two key species, i.e., *Leymus chinensis* and *Phragmites communis*, were selected.

## Materials and Methods

### Site description

This research was conducted at the Songnen Grassland Ecological Research Station (44°45′N, 123°45′E), Northeast Normal University, Jilin Province, northeastern China. The mean annual precipitation is approximately 400 mm, 90% of which occurs from May to October. The mean annual air temperature is 4.9°C, and the mean annual land surface temperature is 6.2°C The soil in the study area is a sodic saline meadow soil with a pH of 8.2 and 3–4% organic matter in the surface soil (0–20 cm). The vegetation in the experimental site is dominated by *Leymus chinensis*, *Kalimeris integrifolia*, *Phragmites communis* and *Carex duriuscula*. The growing season of vegetation is from May to September.

### Experimental design

We used a complete randomized block factorial experimental design with two factors: warming and N addition. There were four treatments: control (C), warming (W), N addition (N), and warming plus N addition (W+N) with 6 replications per treatment. The size of each plot was 2 m × 3 m. All of the warmed plots were heated continuously using infrared radiators (MSR-2420, Kalglo Electronics Inc. Bethlehem, PA, USA) suspended at a height of 2.25 m over the center of the plot. The heat waves of the infrared radiator are identical to the sun's energy, and the effects of the infrared heaters on the soil temperature were spatially uniform [29]. In each control or N addition plot, one ‘dummy’ heater with the same shape and size was installed to imitate the shading effects of the infrared radiator. All of the heaters in the warming treatments were set at a radiation output of approximately 1700 W. It is estimated that in China anthropogenic N deposition has increased from 0.8 g m^-2^ yr^-1^ to 2.1 g m^-2^ yr^-1^, and which can reach 8–9 g N m^-2^ yr^-1^ in central China, and even higher N deposition is expected in the future owing to land-use change and activities [20,30]. In the northern temperate grassland ecosystem, the community saturation resulting from N deposition was approximately 10.5 g m^-2^ yr^-1^ [27], even though atmospheric N deposition in this area was only 2.7 g m^-2^ yr ^-1^ in the last decade [31]. Thus, in the N addition treatments plots, ammonium nitrate (10 g m^-2^ yr^-1^) was added as a pulse of aqueous N on the first day in May every year. In the control and warming plots, the same amount of water (without N) as the N addition treatment was added to account for N addition-induced differences in water availability. The water we added in the four treatments equal to 10 mm m^-2^. The experiment started in May 2006 and was terminated in September 2009.

### Sampling and chemical analysis

During the middle of August 2008 (at the most productive season of plant growth), we randomly selected 10 plants of *L*. *chinensis* and *P*. *communis*, each plant collected five fully expanded green leaves in total 50 leaves between 10:00–11:00 am within each plot, and the rhizosphere soil of *L*. *chinensis* and *P*. *communis* was collected for nutrient analysis. The selected whole leaves were cut into pieces and placed in a test tube which contains 10 ml extractant (80% acetone and absolute alcohol at a ratio 1:1). The test tubes were incubated at 70°C for 30 min [32], and then cooled in the dark. The cooled extract was analyzed using a spectrophotometer (UV-2201, Essentia, Japan) at 440, 649 and 665 nm. The concentrations of chlorophyll *a* (Chl *a*), chlorophyll *b* (Chl *b*), and carotenoid were calculated according to the equation proposed by Wellburn [33].

Soluble sugars were extracted according to the methods described by Jin et al. [34]. Leaves were sampled between 10:00–11:00 am, oven-dried at 65°C for 48 h, and ground. Approximately 100 mg of dry leaf powder of each sample was extracted using 80% ethanol (v/v) at 80°C for 40 min, and then the extracts were centrifuged at 12,000 g for 15 min. This step repeated three times. The three extracts were mixed, and then subjected to soluble sugar analysis using the anthrone-sulfuric acid method [35]. Sucrose was determined according to the method presented by Liao and Yu [36]. Extracts (0.5 ml) from the previous step were placed in glass vials containing 3.5 ml of 0.1% (v/v) m-dihydroxybenzene and 3.5 ml of 30% (v/v) HCl, heated at 80°C for 10 min, and absorbance was determined at 480 nm using a spectrophotometer. NaOH (0.2 ml of 2 mol L^-1^) was added to the 0.5-ml extracts after hot ethanol extraction, heated for 2 min, added to 3.5 ml of 0.1% (v/v) m-dihydroxybenzene and 3.5 ml of 10% (v/v) HCl, heated at 80°C for 10 min, and then the absorbance of fructose was determined at 480 nm using a spectrophotometer (UV-2201).

The leaves of the two species were dried at 65°C for 48 h, and then ground using a ball mill. The total C content was measured using the H_2_SO_4_–K_2_Cr_2_O_7_ oxidation method [37]. Subsamples were digested in H_2_SO_4_–H_2_O_2_ [38]. Total N contents were determined using an Alpkem autoanalyzer (Kjeltec System 2300 Foss Tecator, Sweden). Total P was measured colorimetrically at 880 nm after reaction with molybdenum blue.

### Soil microclimate and nutrient measurements

Soil temperature and moisture were measured using an ECH_2_O dielectric aquameter (EM50/R, USA). One EM50/R probe (Decagon Ltd, Pullman WA, USA) was buried at a depth of 15 cm in each experimental plot, and soil temperature (ST) and soil moisture (SM) were measured at one-hour intervals. Soil total N was measured using the Kjeldahl method. Soil available P content was determined using NaHCO_3_ extraction and a molybdenum blue colorimetric method using UV photometry (UV-2201, Essentia, Japan) at 660 nm. The plant cover the cover was estimated using a modified point-frame method [39], plant species *L*. *chinensis* and *P*. *communis* were cut along the surface and dried at 65°C for 48 h, and then recorded plant aboveground biomass.

### Statistical analysis

All statistical analyses were performed using SPSS 16.0 (SPSS Inc., Chicago, IL, USA). Four-way ANOVAs was used to test the effects of block, warming, N addition and species on leaf chlorophyll, soluble sugars, plant nutrient concentrations and stoichiometric ratios. Significant differences between treatment means were analyzed using Tukey’s post-hoc multiple comparison test after one-way ANOVAs. Statistical significance was determined at a level of *P* = 0.05.

## Results

### Soil microclimate and nutrients

**Fig 1:**
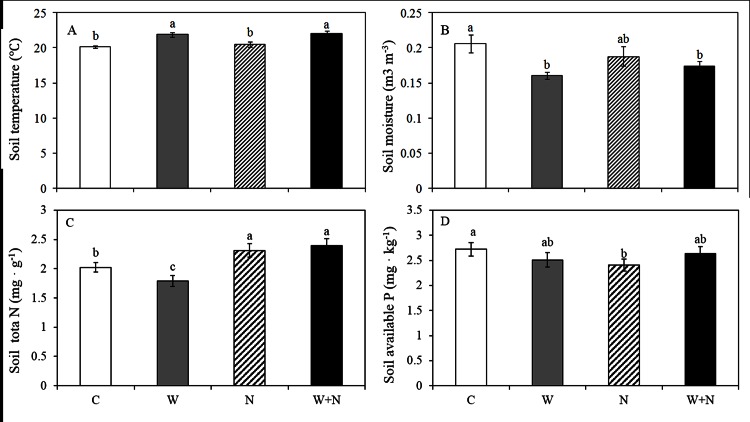
**The effects of warming and nitrogen addition on soil temperature (A), soil moisture (B), soil total N concentration (C) and soil available P concentration (D).** Treatments are as follows: C, control; W, warming; N, nitrogen addition; W+N, both warming and N addition. Different lowercase letters represent significant difference among different treatments at 0.05 level. Data are adjusted means ±SE.

Compared with the control treatment, the warming and warming plus N addition treatments increased ST by 1.7°C (*P*<0.05) and 1.9°C (*P*<0.05), respectively; N addition had no impact on ST (*P*>0.05, Fig 1A). The warming and warming plus N addition treatments reduced the mean SM by 22.1% (*P*<0.05) and 15.4% (*P*<0.05), respectively, compared with the control treatment (Fig 1B). N addition did not affect SM (*P*>0.05). Warming did not affect the soil N content (*P*>0.05), but N addition and the warming plus N addition treatments increased the soil N content by 34.1% (*P*<0.05) and 38.7% (*P*<0.05, Fig 1C), respectively. N addition reduced the soil P content by 11.6% (*P*<0.05) compared with the control treatment, while warming and warming plus N addition had no impact on the soil P content (*P*<0.05, Fig 1D).

### Photosynthetic pigment content

**Fig 2:**
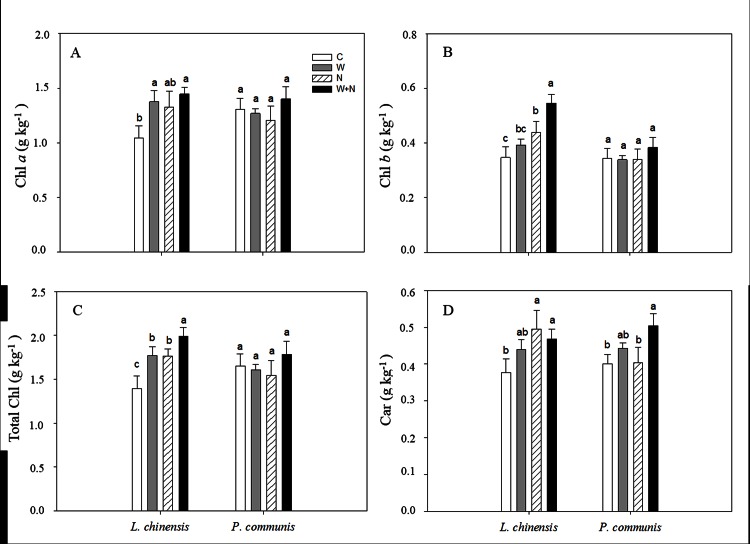
**The effects of warming and nitrogen addition on foliar Chl *a* (A), Chl *b* (B), Total Chl (C) concentration, Car (D).** Treatments are as follows: C, control; W, warming; N, nitrogen addition; W+N, both warming and N addition. Different lowercase letters represent significant difference among different treatments at 0.05 level. Data are adjusted means ±SE.

Warming significantly increased the Chl *a* and total Chl (*a*+*b*) contents of *L*. *chinensis* (*P* <0.05; Fig 2A and 2C), but had no effect on the Chl *b* and carotenoid contents (Fig 2B and 2D). N addition caused a significant increase in the Chl *b*, total Chl (*a*+*b*) and carotenoid contents of *L*. *chinensis* (*P*<0.05; Fig 2B and 2D), but had no impact on Chl *a* (*P* = 0.057). Neither warming nor N addition had an impact on the Chl *a*, Chl *b*, total Chl and carotenoid contents of *P*. *communis* (Fig 2). Significant interactive effects of warming × N addition on the Chl *a*, total Chl (*a*+*b*) and carotenoid contents were observed (Table 1). Significant interactive effect of species × warming on the Chl *b* and carotenoid contents (*P*<0.001, Table 1) and significant interactive effect of species × N addition on the Chl *b* content (*P*<0.001, Table 1) were observed.

**Table 1:** Results of four-way ANOVAs on the effects of species identity (S), warming (W), nitrogen addition (N) and their interactions on plant biomass, cover, leaf chlorophyll, carotenoids and soluble sugars.

	Cover	Biomass	Chl *a*	Chl *b*	Total Chl	Car	Sucrose	Fructose	Total Soluble Sugar
Block	ns	ns	ns	ns	ns	ns	ns	ns	ns
S	**	*	***	***	***	***	***	*	***
W	ns	*	ns	***	ns	***	ns	ns	ns
N	*	**	ns	**	*	ns	**	ns	*
S × W	ns	*	ns	***	ns	***	ns	ns	*
S × N	*	**	ns	***	ns	ns	*	ns	ns
W × N	ns	*	**	ns	**	***	ns	ns	ns
S × W × N	ns	*	ns	ns	ns	*	***	ns	ns

*P<0.05

**P<0.01

***P<0.001

ns indicates no significant difference.

### Soluble sugar

**Fig 3:**
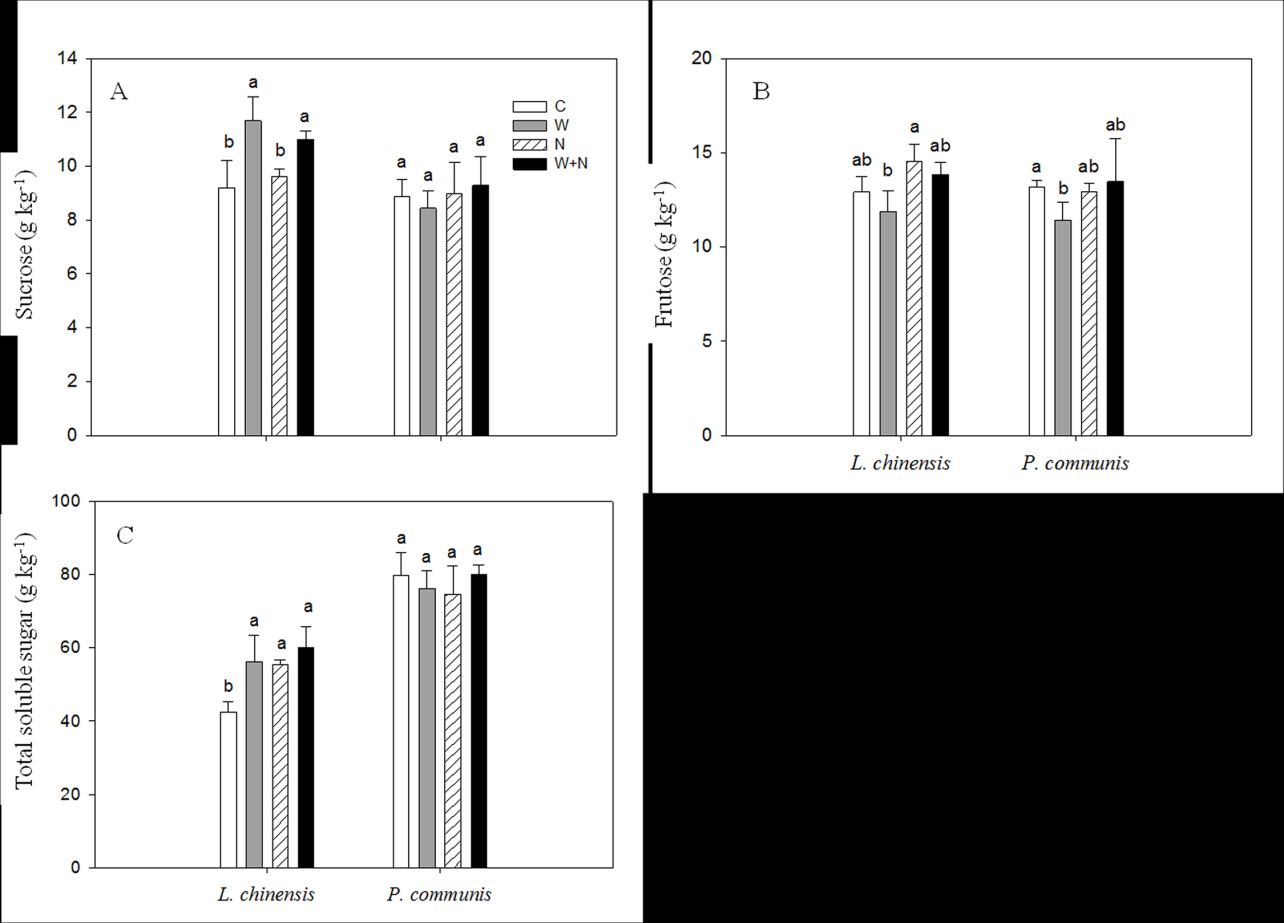
**The effects of warming and nitrogen addition on sucrose (A), fructose (B), Total soluble sugar (C) in plant leaves.** Treatments are as follows: C, control; W, warming; N, nitrogen addition; W+N, both warming and N addition. Different lowercase letters represent significant difference among different treatments at 0.05 level. Data are adjusted means ±SE.

Warming increased the sucrose and total soluble sugar contents of *L*. *chinensis* by 27.0% (*P*<0.05; Fig 3A) and 36.5% (*P*<0.05; Fig 3C), respectively, but had no influence on the fructose content (*P* = 0.15; Fig 3B). N addition increased the total soluble sugar content of *L*. *chinensis* by 30.4% (*P*<0.05; Fig 3C) but had no impact on the sucrose and fructose contents (*P*>0.05; Fig 3A and 3B). Neither the warming nor the N addition treatments affected the sucrose, fructose or total soluble sugar contents of *P*. *communis* (Fig 3). This species only responded to N addition with respect to sucrose, and responded to warming with respect to the total soluble sugar content (Table 1). Significant interactive effect of warming × N addition on the sucrose content was detected (Table 1).

### Foliar stoichiometric ratios

**Fig 4:**
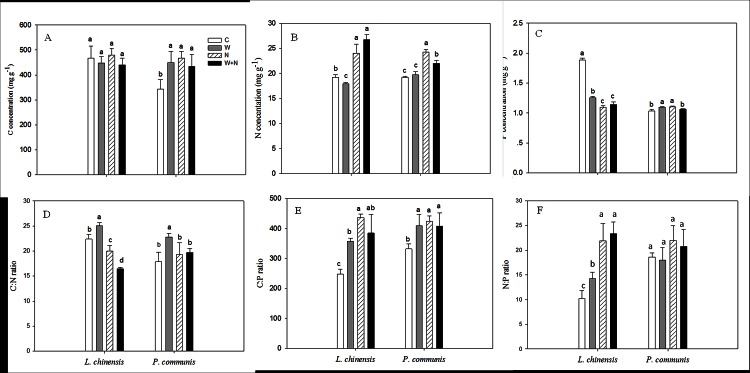
**The effects of warming and nitrogen addition on foliar carbon (A), nitrogen (B), phosphorus (C) concentration, C:N (D), C:P (E) and N:P ratio (F).** Treatments are as follows: C, control; W, warming; N, nitrogen addition; W+N, both warming and N addition. Different lowercase letters represent significant difference among different treatments at 0.05 level. Data are adjusted means ±SE.

Warming and N addition did not affect the C concentration of *L*. *chinensis* (*P*>0.05; Fig 4A), but warming decreased the foliar N and P concentrations by 6.7% (*P*<0.05; Fig 4B) and 33.5% (*P*<0.01; Fig 4C) than control, respectively. Warming increased the C:N (*P*<0.05; Fig 4D), C:P (*P*<0.01; Fig 4E) and N:P ratios (*P*<0.05; Fig 4F) of *L*. *chinensis*. In contrast, warming stimulated the foliar C (*P*<0.05; Fig 4A) and P (*P*<0.05; Fig 4B) concentrations of *P*. *communis*, and the C:N and C:P ratios increased by 27.6% (*P*<0.05; Fig 4D) and 23.4% (*P*<0.05; Fig 4E), respectively. N addition increased the C concentration of *P*. *communis*, but had no impact on *L*. *chinensis*. N addition significantly increased the N concentration in both species (*P*<0.05; Fig 4B). Compared to control, N addition decreased the P concentration of *L*. *chinensis* (*P*<0.05; Fig 4C) but increased the P concentration of *P*. *communis* (*P*<0.05; Fig 4C), respectively. N addition reduced the C:N ratio of *L*. *chinensis* by 18.1% (*P*<0.05; Fig 4D), but increased the C:P ratios of *L*. *chinensis* and *P*. *communis* by 76.1% (*P*<0.001) and 26.8% (*P*<0.05), respectively. Furthermore, N addition significantly increased the N:P ratio of *L*. *chinensis* (*P*<0.01), but had no influence on *P*. *communis* (*P* = 0.062; Fig 4F). There were no interactive effects of warming × N addition on the concentrations of C, N, and P, and the C:N, C:P, N:P ratios of both species (Table 2). Significant interactive effect of species × warming on the C:N and N:P ratios, and significant interactive effect of species × N addition on the C and N concentrations were observed (Table 2).

**Table 2:** Results of four-way ANOVAs on the effects of species identity (S), warming (W), nitrogen addition (N) and their interactions on leaf nutrient concentration and stoichiometric ratios.

	C	N	P	C:N	C:P	N:P
Block	ns	ns	ns	ns	ns	ns
S	***	***	***	***	**	***
W	**	ns	ns	*	ns	*
N	***	***	*	***	ns	*
S × W	ns	ns	ns	*	ns	*
S × N	*	***	ns	ns	ns	ns
W × N	ns	ns	ns	ns	ns	ns
S × W × N	ns	ns	ns	ns	ns	ns

*P<0.05

**P<0.01

***P<0.001

ns indicates no significant difference.

## Discussion

### Effects of warming and N addition on photosynthetic pigments

Chlorophyll is the most important photosynthetic pigment. The chlorophyll content can determine photosynthesis and plant growth. A few studies reported that elevated temperature did not affect or decreased [40–41] the plant chlorophyll concentration. Previous study found that warming significantly increased the chlorophyll contents of *Picea asperata* and *Pinus tabulaeformis* [11]. In the current study, warming greatly increased the chlorophyll content of *L*. *chinensis* (Fig 2A and 2C), which is consistent with previous results from forest ecosystem [11]. The increased chlorophyll content in *L*. *chinensis* caused by warming was probably due to increased leaf photosynthesis and photosynthetic products [4], however, warming did not alter the chlorophyll content of *P*. *communis* in this study, reflecting notable variation in responses among plant species to warming. Shen et al. reported that warming decreased the carotenoid content of *G*. *straminea* [41]; in the present study, warming did not affect the carotenoid content of either species.

In a previous study, N fertilization did not affect the chlorophyll content of *L*. *chinensis* under a clipped defoliation condition [42]. In the present study, N addition did not affect the chlorophyll and carotenoid contents of *P*. *communis*, which is consistent with the above results. However, nitrogen addition caused a significant increase in the chlorophyll and carotenoid contents of *L*. *chinensis*, which is in agreement with previous results [43]. The increase in chlorophyll concentration might be induced by the improved growth of *L*. *chinensis* in response to higher N availability because N addition could further increase soil N availability in the Songnen grassland [5]. Moreover, the increase of chlorophyll suggest that N addition improved the plant photosynthesis and would improve plant growth, in fact, N addition increased plant cover and aboveground biomass of *L*. *chinensis*, but had no impact on *P*. *communis* (S1 and S2 Figs). The interactive effects of warming × N addition resulted in greater chlorophyll accumulation in *L*. *chinensis* compared with warming or N addition alone (Fig 2). Although neither N addition nor warming did not alter the chlorophyll content of *P*. *communis*, there was a significant interactive effect of warming × N addition on the carotenoid content of *P*. *communis*, suggesting that this species responded modestly to the changes in soil moisture caused by warming and soil nutrient availability resulting from N addition. The results suggest that changes in multi-climate factors might have a greater effect on the photosynthesis of terrestrial plants than individual factors.

### Effects of warming and N addition on soluble sugars

Soluble sugar is one of the most important osmotic adjustment substances in plant tissue and plays an important role in anti-adversity of plants [44–45]. Warming significantly increased the total soluble sugar and sucrose contents of *L*. *chinensis*, which is in accordance with previous results [40]. The increase in soluble sugars in *L*. *chinensis* might decrease leaf water potential, can prevent plant drought stress, and can reduce *L*. *chinensis* damage caused by increased temperature. The result suggests that *L*. *chinensis* might resist warming by increasing the synthesis of soluble sugar. Warming did not affect the sucrose and total soluble sugar contents of *P*. *communis* but reduced the fructose content. The result might be related to the higher warming tolerance of *P*. *communis* because the deep root which can reach 1 m and help uptake of water [46], and reduce the effects of warming on the soluble sugar synthesis. Whereas, most root of *L*. *chinensis* distribute in 0–20 cm soil [47], so that *L*. *chinensis* might increase the soluble sugar to reduce the negative effect of warming on plant growth due to the reduction of soil moisture caused by warming.

However, the effect of N addition on soluble sugars was not consistent, and several studies have found that N addition significantly decreased soluble sugars [43–44]; there is a previous study reported that an increase in the soluble sugar content in response to N addition [48]. In the present study, N addition significantly increased the total soluble sugar in leaf of *L*. *chinensis*, suggesting that N input altered organic carbon allocation and resulted in higher photosynthetic production. N addition had no impact on the soluble sugars of *P*. *communis*, which might be related to the root morphological characteristics of this species. The roots of *P*. *communis* grow deeper [46] than those of *L*. *chinensis* [47]. Consequently, the small increase in N and warming treatments did not affect the photosynthetic pigments and products of *P*. *communis*. There were no interactive effects of warming × N addition on the soluble sugars of either species.

Furthermore, although *L*. *chinensis* reduced the negative effect of warming on plant growth by increasing total chlorophyll and soluble sugars, aboveground biomass significantly decreased (S2 Fig). This result suggests that if the warming will continue in the future, the dominance of *L*. *chinensis* would decline and the dominance of *P*. *communis* would increase accordingly. If the N deposition will continue to increase the dominance of *L*. *chinensis* would enhance and the dominance of *P*. *communis* would decline accordingly in the future, which will alter plant community composition as previous study [28]. However, the simultaneous warming and N addition might alleviate influence of warming and N addition alone on plant growth (S1 and S2 Figs) and alter plant community composition, which is consistent with our early study [28].

### Effects of warming and N addition on foliar stoichiometric ratios

The elemental stoichiometry of plants is related to a variety of processes that can reflect multiple plant responses to changes in the biotic and abiotic environment [12, 49]. For instance, the changes of N/P ratio in leaves can affect metabolically active and the reduction of N/P ratio can increase P allocation to RNA [50]. Both of the grass species examined in this study showed a strong response to warming and N addition with respect to the N and P concentrations and their ratio (Fig 4). Elevated temperature did not affect the N concentration or C:N ratio of *Festuca arundinacea* and *Dactylis glomerata* [51] but continue to increase the foliar P concentration of *Erica multiflora* [52]. In our present study, warming significantly decreased the N and P concentrations of *L*. *chinensis* and increased the C:P and N:P ratios, these results are in agreement with the results from other studies [12,53,54]. The results support that warming reduced the amount of nutrients invested to produce proteins to sustain biochemistry reactions [55]. Moreover, it is possible that warming decreased the soil water content and reduced the availability of N and P (Fig 1C and 1D), which led to a decrease in the uptake of these nutrients from the soil. However, the responses of the C and P concentrations of *P*. *communis* to warming were opposite to those of *L*. *chinensis*, i.e., the C and P concentrations and the C:N, C:P ratios of *P*. *communis* increased in response to warming. We hypothesize that this might be related to the root morphological characteristics of *P*. *communis*, e.g., warming reduced the soil water content of the surface soil (Fig 1B), which resulted in deeper root growth for greater nutrient absorption.

N enrichment can increase plant N concentration and reduce the C:N ratio [56]. In the current study, nitrogen addition increased the foliar N concentration and decreased the C:N ratio of *L*. *chinensis*, but had no impact on the C concentration. N addition significantly increased the foliar P concentration of *P*. *communis*, which suggests that N addition might enhance the activities of soil phosphatase and improve P uptake in grassland ecosystems. The observed higher foliar N:P ratios of both species in fertilized plots may have resulted from the greater increase in N compared with P concentration in response to N addition, which is consistent with previous studies [26,57]. There were no interactive effects of warming × N addition on the leaf nutrient concentrations and stoichiometric ratios of either species (Table 2). The results suggest that responses of different species to warming and N addition should be considered carefully in future.

### Conclusions and implications

In conclusion, our study shows that both N addition and warming have direct effects on photosynthetic products and foliar stoichiometric variables. Increases in the photosynthetic pigments, carotenoids and soluble sugars of *L*. *chinensis* following warming and N addition indicate that climate changes (warming and N addition) may stimulate plant photosynthesis and the accumulation of photosynthetic products. Our results showed that N addition significantly increased the foliar N concentration and C:P ratio of the two species, indicating that N input might stimulate plant growth and accelerate litter decomposition in the studied ecosystem. Warming can also alter the foliar elemental stoichiometry, but the responses of these two species to warming were significantly different. Warming and N addition interacted to affect the photosynthetic pigments of the two species, but had no interactive effect on foliar stoichiometry. The photosynthetic products and foliar stoichiometry of these species in response to warming and N addition showed inconsistent results, which suggests that clearly determining the influence of warming and N addition on plant physiological characteristics is important to fully understand the mechanism through which climate changes alter plant community composition in grasslands.

## Supporting Information

S1 Fig **The effect of warming and N addition on cover of species *Leymus chinensis* (A) and *Phragmites communis* (B).** Treatments are as follows: C, control; W, warming; N, nitrogen addition; W+N, both warming and N addition. Data are adjusted means ±SE.
(TIF)

S2 Fig **The effect of warming and N addition on aboveground biomass of species *Leymus chinensis* (A) and *Phragmites communis* (B).** Treatments are as follows: C, control; W, warming; N, nitrogen addition; W+N, both warming and N addition. Data are adjusted means ±SE.
(TIF)

S1 Table Results of four-way ANOVAs on the effects of species identity (S), warming (W), nitrogen addition (N) and their interactions on plant biomass, cover, leaf chlorophyll, carotenoids and soluble sugars. (DOCX)

S2 Table Results of four-way ANOVAs on the effects of species identity (S), warming (W), nitrogen addition (N) and their interactions on leaf nutrient concentration and stoichiometric ratios. (DOCX)

S3 Table The effects of warming and nitrogen addition on foliar Chl *a* (A), Chl *b* (B), Total Chl (C) concentration, Car (D).
(DOCX)

S4 Table The effects of warming and nitrogen addition on sucrose (A), fructose (B), Total soluble sugar (C) in plant leaves.
(DOCX)

S5 Table The effects of warming and nitrogen addition on foliar carbon (A), nitrogen (B), phosphorus (C) concentration, C:N (D), C:P (E) and N:P ratio (F).
(DOCX)
