# Correction: Warming and Nitrogen Addition Alter Photosynthetic Pigments, Sugars and Nutrients in a Temperate Meadow Ecosystem

**DOI:** 10.1371/journal.pone.0158249

**Published:** 2016-06-21

**Authors:** Tao Zhang, Shaobo Yang, Rui Guo, Jixun Guo

There are errors in the Funding section. The funding for this paper is as follows: This work was funded by the National Natural Science Foundation of China (31300097 and 31470405) and the Strategic Priority Research Program of the Chinese Academy of Sciences (XDA05050601-01-19).
