# Supplementary figures and images for: Warming and Nitrogen Addition Alter Photosynthetic Pigments, Sugars and Nutrients in a Temperate Meadow Ecosystem
Source: PLoS One. 2016 May 12;11(5):e0155375. doi: 10.1371/journal.pone.0155375 (PMC4865211; doi:10.1371/journal.pone.0155375)

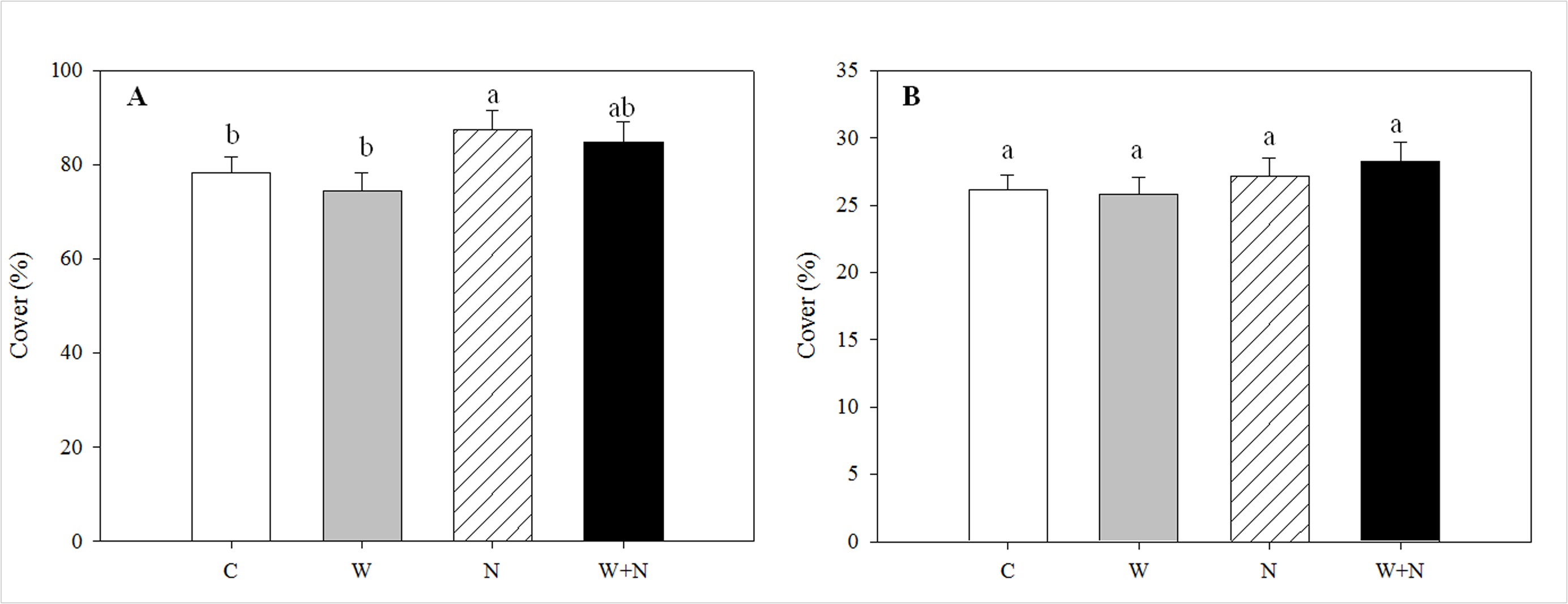

Supplement: S1 Fig — The effect of warming and N addition on cover of species Leymus chinensis (A) and Phragmites communis (B). Treatments are as follows: C, control; W, warming; N, nitrogen addition; W+N, both warming and N addition. Data are adjusted means ±SE. (TIF) [file pone.0155375.s001.tif]

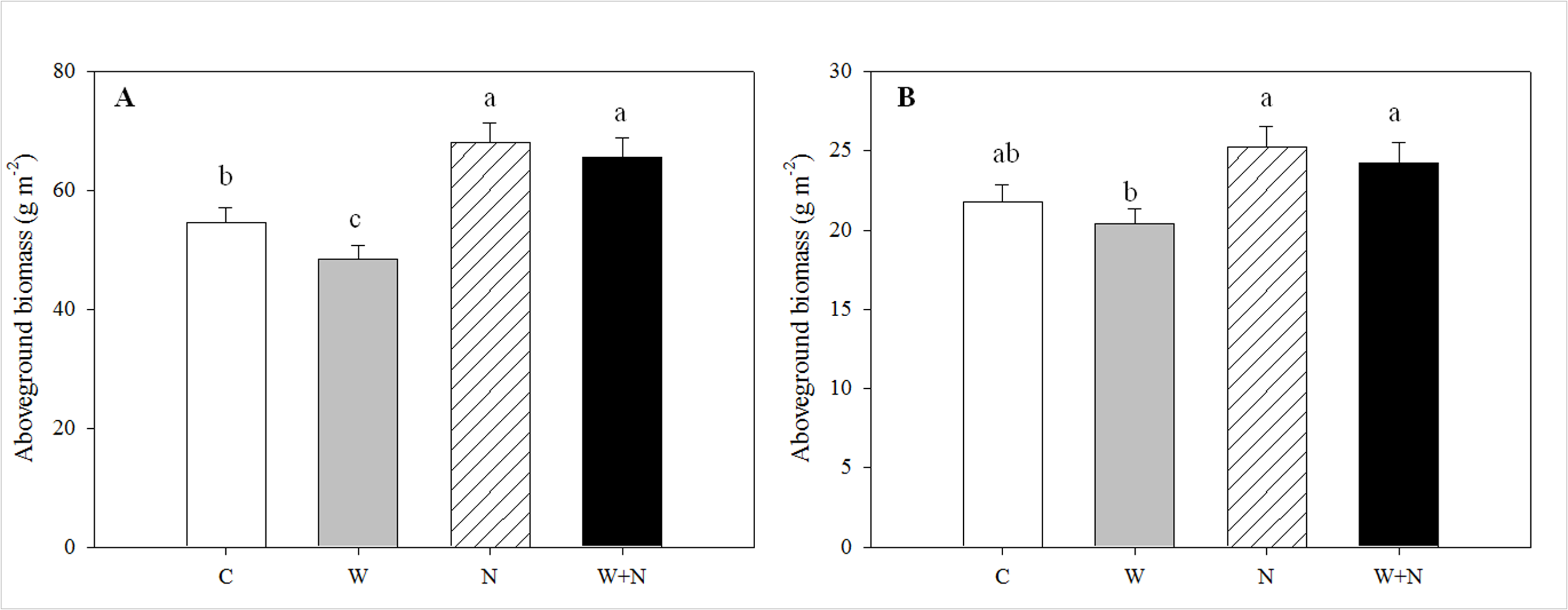

Supplement: S2 Fig — The effect of warming and N addition on aboveground biomass of species Leymus chinensis (A) and Phragmites communis (B). Treatments are as follows: C, control; W, warming; N, nitrogen addition; W+N, both warming and N addition. Data are adjusted means ±SE. (TIF) [file pone.0155375.s002.tif]
