# Supplementary material for: Warming and Nitrogen Addition Alter Photosynthetic Pigments, Sugars and Nutrients in a Temperate Meadow Ecosystem
Source: PLoS One. 2016 May 12;11(5):e0155375. doi: 10.1371/journal.pone.0155375 (PMC4865211; doi:10.1371/journal.pone.0155375)
Supplement: S1 Table — (DOCX) [file pone.0155375.s003.docx]

**Spport Information**

**S1 Table** **Results of four-way ANOVAs on the effects of species identity (S), warming (W), nitrogen addition (N) and their interactions on plant biomass, cover, leaf chlorophyll, carotenoids and soluble sugars.**

|  | Cover | Biomass | Chl *a* | Chl *b* | Total Chl | Car | Sucrose | Fructose | Total Soluble Sugar |
| --- | --- | --- | --- | --- | --- | --- | --- | --- | --- |
| Block | ns | ns | ns | ns | ns | ns | ns | ns | Ns |
| Species (S) | ** | * | *** | *** | *** | *** | *** | * | *** |
| W | ns | * | ns | *** | ns | *** | ns | ns | Ns |
| N | * | ** | ns | ** | * | ns | ** | ns | * |
| S×W | ns | * | ns | *** | ns | *** | ns | ns | * |
| S×N | * | ** | ns | *** | ns | ns | * | ns | Ns |
| W×N | ns | * | ** | ns | ** | *** | ns | ns | Ns |
| S×W×N | ns | * | ns | ns | ns | * | *** | ns | Ns |

**P*<0.05; ***P*<0.01; ****P*<0.001; ns indicates no significant difference.
