# Supplementary material for: Warming and Nitrogen Addition Alter Photosynthetic Pigments, Sugars and Nutrients in a Temperate Meadow Ecosystem
Source: PLoS One. 2016 May 12;11(5):e0155375. doi: 10.1371/journal.pone.0155375 (PMC4865211; doi:10.1371/journal.pone.0155375)
Supplement: S2 Table — (DOCX) [file pone.0155375.s004.docx]

**S2 Table** **Results of four-way ANOVAs on the effects of species identity (S), warming (W), nitrogen addition (N) and their interactions on leaf nutrient concentration and stoichiometric ratios.**

|  | C | N | P | C:N | C:P | N:P |
| --- | --- | --- | --- | --- | --- | --- |
| Block | ns | ns | ns | ns | ns | Ns |
| Species (S) | *** | *** | *** | *** | ** | *** |
| W | ** | ns | ns | * | ns | * |
| N | *** | *** | * | *** | ns | * |
| S × W | ns | ns | ns | * | ns | * |
| S × N | * | *** | ns | ns | ns | Ns |
| W × N | ns | ns | ns | ns | ns | Ns |
| S × W × N | ns | ns | ns | ns | ns | Ns |

**P*<0.05; ***P*<0.01; ****P*<0.001; ns indicates no significant difference.
