# Supplementary material for: Warming and Nitrogen Addition Alter Photosynthetic Pigments, Sugars and Nutrients in a Temperate Meadow Ecosystem
Source: PLoS One. 2016 May 12;11(5):e0155375. doi: 10.1371/journal.pone.0155375 (PMC4865211; doi:10.1371/journal.pone.0155375)
Supplement: S3 Table — The effects of warming and nitrogen addition on foliar Chl a (A), Chl b (B), Total Chl (C) concentration, Car (D). (DOCX) [file pone.0155375.s005.docx]

**S3 Table The effects of warming and nitrogen addition on foliar Chl *a* (A), Chl *b* (B), Total Chl (C) concentration, Car (D).**

| Treatments | Chl a (g·kg-1) | | Chl b (g·kg-1) | | Chl a +b (g·kg-1) | | Car (g·kg^-1^) | |
| --- | --- | --- | --- | --- | --- | --- | --- | --- |
|  | *L. chinensis* | *P. communis* | *L. chinensis* | *P. communis* | *L. chinensis* | *P. communis* | *L. chinensis* | *P. communis* |
| C | 1.05(0.11) | 1.31(0.10) | 0.35(0.04) | 0.34(0.03) | 1.39(0.15) | 1.65(0.14) | 0.38(0.04) | 0.40(0.03) |
| W | 1.38(0.20) | 1.27(0.05) | 0.39(0.02) | 0.34(0.01) | 1.77(0.10) | 1.61(0.06) | 0.44(0.03) | 0.44(0.01) |
| N | 1.33(0.15) | 1.21(0.13) | 0.44(0.04) | 0.34(0.04) | 1.76(0.08) | 1.55(0.17) | 0.50(0.05) | 0.41(0.04) |
| W+N | 1.45(0.06) | 1.40(0.11) | 0.55(0.03) | 0.38(0.04) | 1.99(0.09) | 1.79(0.15) | 0.47(0.03) | 0.50(0.03) |

Treatments are as follows: C, control; W, warming; N, nitrogen addition; W+N, both warming and N addition. Different lowercase letters represent significant difference among different treatments at 0.05 level. Data are adjusted means with SE in brackets.
