# Supplementary material for: Warming and Nitrogen Addition Alter Photosynthetic Pigments, Sugars and Nutrients in a Temperate Meadow Ecosystem
Source: PLoS One. 2016 May 12;11(5):e0155375. doi: 10.1371/journal.pone.0155375 (PMC4865211; doi:10.1371/journal.pone.0155375)
Supplement: S4 Table — The effects of warming and nitrogen addition on sucrose (A), fructose (B), Total soluble sugar (C) in plant leaves. (DOCX) [file pone.0155375.s006.docx]

**S4 Table** **The effects of warming and nitrogen addition on sucrose (A), fructose (B), Total soluble sugar (C) in plant leaves.**

| Treatments | Sucrose (g·kg^-1^) | | Fructose (g·kg^-1^) | | Total soluble sugar (g·kg^-1^) | |
| --- | --- | --- | --- | --- | --- | --- |
|  | *L. chinensis* | *P. communis* | *L. chinensis* | *P. communis* | *L. chinensis* | *P. communis* |
| C | 42.4(3.0) | 79.7(6.2) | 12.9(0.8) | 13.2(0.3) | 9.2(1.0) | 8.9(0.7) |
| W | 56.2(7.3) | 76.1(4.9) | 11.9(1.1) | 11.4(0.9) | 11.7(0.9) | 8.4(0.7) |
| N | 55.3(1.5) | 74.6(7.7) | 14.5(0.9) | 12.9(0.5) | 9.6(0.3) | 8.9(1.2) |
| W+N | 60.0(5.9) | 80.1(2.5) | 13.8(0.6) | 13.5(2.3) | 11.0(0.3) | 9.3(1.1) |

Treatments are as follows: C, control; W, warming; N, nitrogen addition; W+N, both warming and N addition. Different lowercase letters represent significant difference among different treatments at 0.05 level. Data are adjusted means with SE in brackets.
