# Supplementary material for: Warming and Nitrogen Addition Alter Photosynthetic Pigments, Sugars and Nutrients in a Temperate Meadow Ecosystem
Source: PLoS One. 2016 May 12;11(5):e0155375. doi: 10.1371/journal.pone.0155375 (PMC4865211; doi:10.1371/journal.pone.0155375)
Supplement: S5 Table — The effects of warming and nitrogen addition on foliar carbon (A), nitrogen (B), phosphorus (C) concentration, C:N (D), C:P (E) and N:P ratio (F). (DOCX) [file pone.0155375.s007.docx]

**S5 Table The effects of warming and nitrogen addition on foliar carbon (A), nitrogen (B), phosphorus (C) concentration, C:N (D), C:P (E) and N:P ratio (F).**

| Treatments | C |  | N |  | P |  | C/N |  | C/P |  | N/P |  |
| --- | --- | --- | --- | --- | --- | --- | --- | --- | --- | --- | --- | --- |
|  | *L. chinensis* | *P. communis* | *L. chinensis* | *P. communis* | *L. chinensis* | *P. communis* | *L. chinensis* | *P. communis* | *L. chinensis* | *P. communis* | *L. chinensis* | *P. communis* |
| C | 468(47) | 343(38) | 19.2(0.5) | 19.2(0.2) | 1.89(0.03) | 1.03(0.02) | 24.38(0.94) | 17.86(1.90) | 248.14(15.67) | 332.04(16.52) | 10.18(1.67) | 18.59(0.87) |
| W | 448(25) | 449(45) | 17.9(0.3) | 19.7(0.6) | 1.25(0.02) | 1.10(0.01) | 25.03(0.83) | 22.79(0.75) | 357.26(10.42) | 409.67(37.50) | 14.27(1.25) | 17.97(2.50) |
| N | 479(27) | 468(26) | 24.0(1.8) | 24.2(0.6) | 1.10(0.02) | 1.10(0.02) | 19.96(1.5) | 19.34(4.33) | 437.04(11.25) | 424.29(17.33) | 21.89(3.5) | 21.94(3.00) |
| W+N | 440(27) | 434(49) | 26.7(1.0) | 22.0(0.6) | 1.14(0.04) | 1.06(0.01) | 16.48(0.27) | 19.73(0.82) | 384.95(62.79) | 408.27(44.55) | 23.36(2.33) | 20.69(3.45) |

Treatments are as follows: C, control; W, warming; N, nitrogen addition; W+N, both warming and N addition. Different lowercase letters represent significant difference among different treatments at 0.05 level. Data are adjusted means with SE in brackets.
